# How Does the Appeal of Environmental Values Influence Sustainable Entrepreneurial Intention?

**DOI:** 10.3390/ijerph18031070

**Published:** 2021-01-26

**Authors:** Huatao Peng, Bingbing Li, Chen Zhou, Bert M. Sadowski

**Affiliations:** 1 School of Management, Wuhan University of Technology, 122 Luoshi Road, Hongshan District, Wuhan 430070, China; penghuatao@whut.edu.cn (H.P.); chen.zhou@whut.edu.cn (C.Z.); 2 School of Innovation Sciences, University of Technology Eindhoven, 5612AZ Eindhoven, 8410 Atlas, The Netherlands; b.m.sadowski@tue.nl

**Keywords:** environmental values, sustainable entrepreneurial intention, attitude, social norms, self-efficacy

## Abstract

Global challenges posed by climate change and environmental deterioration are increasingly driving entrepreneurship with sustainable entrepreneurial intention as a key driver in predicting entrepreneurial activities. Together with experience, the environmental values of an entrepreneur are vital for sustainable entrepreneurial intention. However, the extent to which experience is a key factor to start up a sustainable enterprise is still rather unclear. To study the role of experience, we derive from the theory of planned behaviour three factors (personal attitude, social norm and self-efficacy) to examine their impact on environmental values and sustainable entrepreneurial intention. Based on a meta-analysis, the overall directions and effect intensity of the different factors in this relationship can be investigated. We develop a structural equation model to explore the mechanism behind the interaction between the different variables. We utilize information from 37 scientific articles using 40 empirical samples, 117 effect sizes and 192,015 observations. We found that environmental values are indeed positively related to a sustainable entrepreneurial intention. Furthermore, the relationship between environmental values and sustainable entrepreneurial intention is moderated by experience, as well as personal attitude, social norms and self-efficacy. In addition, environmental values are more positively related to the intention to set up a sustainable venture for entrepreneurs with low-experience compared to those entrepreneurs with high-experience. For policy makers and managers, it becomes important to stimulate environmental values to promote sustainable entrepreneurial intentions in order to stimulate the growth of sustainable enterprises. By enhancing these three factors, sustainable entrepreneurial behaviour can be facilitated by increasing entrepreneurs’ sustainable intention.

## 1. Introduction

By responding to globalization and the challenges of climate change, companies are increasingly facing limitations on natural resources and growing costs due to environmental deterioration [1]. Addressing these environmental challenges is becoming an important issue for companies if they want to expand internationally and grow towards environmental sustainability based on sustainable entrepreneurship. To achieve environmental sustainability, entrepreneurship needs to be based upon environmental values (also called “green values” or “ecological values”). There has been some theoretical discussion on the way environmental values affect sustainability entrepreneurship [2]. As new environmental opportunities have emerged due to increasing environmental problems, entrepreneurs have started enterprises that integrate sustainable development into their entrepreneurial plans [3,4]. As sustainable entrepreneurship is often confused with social entrepreneurship and general entrepreneurship, we use the term “sustainable entrepreneurship” in a similar fashion to concepts like “green entrepreneurship” or “ecological entrepreneurship” [5,6]. We define sustainable entrepreneurship as a process in which an entrepreneur realizes his or her intention in building up a company taking into account the enterprise’s sustainability, by organizing this process in a way that a variety of economic, social and ecological angles are addressed [7]. While sustainable entrepreneurial intention can be considered as a cornerstone to transform motivations into behaviour that could predict sustainable entrepreneurial behaviour [8]. In this context, it becomes important to explore the extent to which there is a relationship between environmental values and sustainable entrepreneurial intention.

Environment values increasingly drive entrepreneurs to consider embedding sustainability in their new venture [9]. However, it still is rather unclear how environmental values and sustainable entrepreneurial intention are linked to each other. As entrepreneurs focus on the firms’ economic performance, economic value is usually opposed to social and environmental values [2]. As prices for paying for environmental damages are rising, the intentions for an entrepreneur to set up a sustainable business will be lower as economic profitability might be under pressure [10]. Another line of reasoning has put forward the argument that sustainable entrepreneurs can actually create value by linking to environmental challenges and establish a new start-up business based on sustainability. Environmental values refer to the belief of entrepreneurs for going beyond the traditional economic boundaries of the firm and linking to ecological values [9]. These values and the adoption of sustainable entrepreneurship represent two mechanisms, which are mutually reinforcing [11]. Moreover, sustainable entrepreneurs actually favoured environmental and social values compared to profits [5]. In case of greater environmental damages, which were linked to problems surrounding the acquisition of necessary natural resources, entrepreneurs were rethinking sustainability issues within their business plans and developed an intention to pursue sustainable entrepreneurship [12]. These intentions provided an opportunity for entrepreneurs to start a sustainable enterprise. Therefore, the first objective of the paper is to explore the relationship between environmental values and sustainable entrepreneurial intention. 

As policy makers are increasingly given priority to form strategies related to green practices, sustainability issues are placed at the center of entrepreneurial activities based on environmental values [13]. Entrepreneurial intentions to implement green practices can be considered as way to develop sustainable projects within their firm or to start a new venture. Research has shown that there still is some discussion on the determinants of entrepreneurial intention [14]. Within the theory of planned behaviour (TPB), the intentions of entrepreneurs have been examined in greater detail. Recently, the focus in this tradition has changed and studied the attitudes of actual or nascent entrepreneurs towards sustainability by including their subjective norms and self-efficacy as important predictors when identifying the intentions of entrepreneurs to start up a sustainable enterprise [1,15]. Within the TPB framework, personal intentions towards behaviour (attitude, subjective norms and perceived behavioural control) will be analysed [16]. Previous research has shown in which ways attitudes, subjective norms and self-efficacy of entrepreneurs can be used as predicators affecting the intention to become a sustainable entrepreneur [17]. However, previous scholars used the concept of self-efficacy similar to the notion of perceived behavioural control [18]. In this way, the theory has identified the different dimensions affecting the intention towards entrepreneurship the researcher have, applied the concepts to empirical studies in a variety of countries [19], as well as used these notions in fields, such as the entrepreneurial intentions of students [20] and investors [21]. In addition, the concepts were utilized to study the environmental aspect of value creation and a sustainable start-up intention [2]. In this context, the paper utilizes the TPB framework to explore the relationship between environmental values and sustainable entrepreneurial intention.

Within the TPB framework, personal attitude, social norm and self-efficacy of entrepreneurs are expected to have very different effects on their intention towards integrating sustainability in their companies. For example, even if some studies found that self-efficacy among entrepreneurs appeared to have no significant influence on sustainable entrepreneurial intentions [22], researchers actually found a positive relationship among younger entrepreneurs [22]. Among sustainable entrepreneurs, self-efficacy actually played an import role in the intention towards sustainable entrepreneurs [23]. Furthermore, research has shown that the concept of self-efficacy can be used as a key predictor explaining sustainable entrepreneurial intention [24], as it helps entrepreneurs to build up a green mindset to form a new sustainable firm. Furthermore, research has shown that entrepreneurs need special skills and information to start up a sustainable business, which is related to prior entrepreneurial experiences that are, in turn, affected by entrepreneurial intentions [19]. Entrepreneurs with more business experience have a higher intention to adopt sustainable entrepreneurship [12]. Similarly, students with academic experiences who may be potential entrepreneurs are also more prone to implement sustainable entrepreneurship [9]. As experiences can influence the intention towards sustainable entrepreneurship, this paper studies the extent to which experiences affect sustainable entrepreneurial intention. 

Within the field of environmental studies and within the literature on entrepreneurship, a meta-analysis is already a widely used method to examine the relationship between environmental values and sustainable entrepreneurship [25,26]. Meta-analyses allow researchers to systematically synthesize existing findings of empirical studies, and examine the directions and magnitude of the main effects among different variables [25]. A meta-analysis is able to generalize, even in the event of different empirical outcomes with respect to same relationship [26], and provides researchers with more clarity in assessing these different results in the light of other competing studies. In this context, meta-analysis can generate useful and comprehensive results that are also statically robust [26]. Unfortunately, there has been no recent meta-analysis about the relationship between environmental values and the intention to start a sustainable venture. The third objective of the paper is to fill this void.

With sustainable entrepreneurship emphasizing the sustainability issues surrounding the natural environment in conjunction with addressing social issues and economic goals [27], enterprises are expected to tackle principles of sustainability [10], and consider green values as more important in their behaviour compared to other values [9]. However it currently it is unclear to what extent personal attitudes, social norms and self-efficiency influence entrepreneurial intention [18]. In this context, research needs to address the following questions: (1) How do environmental values influence the intention for sustainable entrepreneurship? (2) To what extent are personal attitudes, social norms and self-efficiency linked to environmental values and sustainable entrepreneurial intention? (3) How does experience affects this relationship. In order to examine the environmental values of entrepreneurs in this context requires researchers to embed them in the current literature on TPB. This paper uses a meta-analysis to explore the relationship between environmental values and sustainable entrepreneurial intention in order to examine the important factors derived from the TPB that influence this relationship.

To study the mechanism by which environmental values influence a sustainable start-up’s intention from the perspective of TPB, this paper utilizes various databases to obtain scientific articles related to the different concepts and analysed the empirical studies on factors related to the intention towards a sustainable start-up, as well as on the relationship among the different factors. In the paper, we used these studies to analyse the links and to construct a structural equations model to explore how to influence these factors related to environmental values and sustainable entrepreneurial intention.

In the following, the paper discusses the theoretical literature on the importance of environmental values for sustainable entrepreneurial intention. Afterwards, it introduces a conceptual model in which the relationship among environmental values and sustainable entrepreneurial intention will be analysed. Furthermore, different factors derived from the theory of planned behaviour are introduced to predict entrepreneurial intention and the empirical results of these factors will discussed. A meta-analytical framework, as widely applied in studies regarding the environment and entrepreneurship, is used to study the effects of these factors on the relationship between environmental values and sustainable entrepreneurial intention.

The structure of the article is as follows. Section 2 reviews the literature and derives the hypotheses. In Section 3, we describe and explain the method of our empirical research, introduce the data sources, the coding of the variables and the procedure of the analysis. Afterwards, we deploy the meta-analysis and a structural equation model to verify the proposed hypotheses. The results of the main empirical results are presented in Section 4. In Section 5, the discussion of the results is presented, and the implications of the research are put forward. Lastly, the limitations of the study and areas for further study are identified in Section 6. 

## 2. Theory and Hypotheses

In the following, we discuss the theoretical foundation of the two related concepts of environmental values and sustainable entrepreneurial intention as well as investigate the different variables affecting these concepts. First, we review the literature on environmental values and sustainable entrepreneurial intention, and then derive eight hypotheses from the literature. 

### 2.1. Environmental Values and Sustainable Entrepreneurial Intention

Values are important predictors for an entrepreneur or a potential entrepreneur’s attitude, perception and behaviour towards sustainable entrepreneurship, as they are related to entrepreneurial intention [19]. Not only do environmental values promote the environmental performance of new ventures, but they are also beneficial for the global ecosystem. An intention to become a sustainable entrepreneur is related to a growing state of consciousness in which entrepreneurs desire to establish a new venture or to establish new core values within an existing organization. Sustainable entrepreneurial intention to start up a company is related to the desire of an entrepreneur to create a new core value [20], in particular environmental values. With growing environmental problems and the depletion of natural resources, entrepreneurs are increasingly driven to adopt an intention towards sustainable entrepreneurship [15]. In addition, entrepreneurs need to be encouraged to pursue sustainable entrepreneurship in order to develop better ways to reduce waste or address problems arising from shortages of natural resources [5]. The development of all kinds of entrepreneurship should rely on the natural environment to produce, survive and live (for example, the raw materials from nature, the air that labourers can breathe), which drives entrepreneurs to focus on environmental sustainability.

Entrepreneurs or potential entrepreneurs tend to depend on their experience when they start up an enterprises [19]. High-level experience helps entrepreneurs or potential entrepreneurs to recognize and attach great importance to sustainable entrepreneurship, which lowers resource costs within their entrepreneurial activities [12]. Values attached to sustainability (including environmental values) require high-level experience in building a start-up; entrepreneurs or potential entrepreneurs should score high on intentions to promote value creation based on sustainability if they want to be successful in the real world [21]. Entrepreneurs with low-level experience are less prone to have high-level environmental values; the impact of these values on sustainable entrepreneurial intention would be rather low. Entrepreneurs or potential entrepreneurs with high-level experience are more likely to use environmental values in allocating resources more efficiently, better arrange working relations and utilize the methods of production and innovation in order to reduce environmental costs. In this way, entrepreneurs can realize objectives related to sustainable entrepreneurship. 

Overall, this logic suggests the following hypotheses:

**Hypothesis** **1a.** *Environmental values will be positively related to sustainable entrepreneurial intention.*

**Hypothesis** **1b.** *The relationship between environment value and sustainable entrepreneurial intention will be moderated by the level of experience.*

### 2.2. Theory of Planned Behaviour and Sustainable Entrepreneurial Intention

TPB has been widely used to predict the intention and behaviour of individuals in a variety of academic fields, including entrepreneurship [28]. TPB has shown that the behaviour of entrepreneurs is the result of entrepreneurial intention; therefore, it could be used to derive factors explaining sustainable entrepreneurship [29]. Strong entrepreneurial intention contributes to many options for different kinds of entrepreneurial behaviour [28]. It also could be used to characterize an intention towards sustainable entrepreneurship as a function of attitude, social norms and self-efficacy [1]. According to previous research [1,9,28], attitude explains the extent to which an entrepreneur is able to evaluate his entrepreneurial behaviour, and use this evaluation to form subsequent entrepreneurial intention [9]. Social norms explain entrepreneurs’ assessment of social pressure to develop certain entrepreneurial behaviour [1]. This behaviour is therefore influenced by the extent to which the social environment reflects on the actions of the entrepreneur [9]. Self-efficacy is defined as the entrepreneur’s judgment of being able or owning enough competence to perform entrepreneurial activities [17]. Under the condition of realizing environmental values, these three variable factors (personal attitude, social norm and self-efficacy) are expected to influence the relationship between environmental values and sustainable entrepreneurial intention in the following way. 

#### 2.2.1. Attitude 

In the face of pressure from environmental degradation, entrepreneurs might be forced to consider environmental protection measures when they intend to start up an enterprise. The expected benefits and advantages related to environmental values drive entrepreneurs to attach some importance to the environmental aspects in value creation within the enterprise [2]. The extent of the implementation of sustainable activities helps entrepreneurs or potential entrepreneurs to identify and create environmental values. The awareness, importance and popularization of value that comes from the environment will help entrepreneurs to adjust their efforts to put sustainable behaviour into practice [12]; it also influences the degree to which sustainable entrepreneurial intention is formed. A more favourable attitude to sustainable entrepreneurship could contribute sustainable initiatives that are much more feasible [1]. A positive attitude constitutes the source of entrepreneurial motivation to entrepreneurial behaviour [23]. Entrepreneurs with a strong attitude tend to act on value creation from the environment, which makes it more possible for intention to take shape and thus to realize sustainable entrepreneurship [9]. Attitude promotes the sustainability of the relationship between environmental values and sustainable entrepreneurial intention.

In a similar vein, experience helps entrepreneurs to easier recognize the consequence of the main relationship, for the reason that environmental factors may be vital catalysts for successful sustainable entrepreneurship [12]. Entrepreneurs or potential entrepreneurs with a strong awareness of green value creation may rely on high-level experience to use scientific and reasonable methods to transform intention to sustainable entrepreneurial behaviour [29]. These conditions quickly shape an attitude towards sustainable entrepreneurship [9], while the entrepreneurs or potential entrepreneurs’ positive and strong attitude obviously being conducive to the formation of a sustainable entrepreneurs’ intention to start a new firm [15]. Based on this logic, we hypothesize:

**Hypothesis** **2a.** *The relationship between environment values and sustainable entrepreneurial intention will be mediated through attitude to sustainable entrepreneurship.*

**Hypothesis** **2b.** *The relationship between environment values and attitude to sustainable entrepreneurship will be positively moderated by the level of experience.*

#### 2.2.2. Social Norms

The pressures of social norms could accelerate sustainable entrepreneurial actions and sustainable values (including importance of environmental values), which are expected to be driven by the pressure of social norms [1]. Based on environmental values, entrepreneurs or potential entrepreneurs pursue sustainable entrepreneurship, as affected by strong social norms [10]. In trying to protect the global environment and facilitating a transition of the company towards a green economy [1], more and more start-up companies are targeting sustainability on the basis of environmental protection and becoming concerned about very specific environmental values [12]. Many entrepreneurs are motivated to support green values for all stakeholders along the supply chain by pursuing a move towards green technologies [13]. An increase in these perceived social norms (or subjective norms) can stimulate entrepreneurs or potential entrepreneurs to strengthen their entrepreneurial intentions. Sustainable entrepreneurial behaviour will not occur if there is no relevant intention [1]. 

Experience allows entrepreneurs or potential entrepreneurs to identify and develop multiple ways to realize their values with respect to environmental protection [12], as well as enables them to include ecological values in their ventures. The advantages of green values are related to improvement in tolerance and acceptance of social norms and they affect the experience of the entrepreneurs or potential entrepreneurs. Greater experience contributes to more importance attached to environmental values and a greater meaning of social norms is related to more sustainable entrepreneurial activities. For example, cooperation driven by high-level social norms related to sustainable practices will affect stakeholders in the supply chain in a way that would draw entrepreneurs’ attention towards higher sustainability [13]. In summary, the following hypotheses can be developed:

**Hypothesis** **3a.** *The relationship between environment values and sustainable entrepreneurial intention will be mediated through social norms.*

**Hypothesis** **3b.** *The relationship between environment values and social norms of entrepreneurs or potential entrepreneurs will be positively moderated by the level of experience.*

#### 2.2.3. Self-Efficacy

Entrepreneurs or potential entrepreneurs’ self-efficacy as a predictor of intention to establish sustainable entrepreneurship is positively influenced by sustainable entrepreneurial intention [7]. Environmental values allow entrepreneurs or potential entrepreneurs to attach a high importance to the development of sustainability within enterprises. Self-efficacy enhances entrepreneurs or potential entrepreneurs’ confidence to transform environmental values into sustainable behaviour. Therefore, self-efficacy gives rise to the ability to realize sustainability, e.g., in cases were energy efficiency has to be achieved [30]. Entrepreneurs or potential entrepreneurs with a higher self-efficacy are more able to effectively use environmental values to generate sustainable activities within the firm [2]. These activities could help reduce the waste of resources, put limitations on the use of remaining resources and energy as well as initiate the development of new technologies and new products to gain in terms of sustainability over the long run. Environmental values could increase the transformation towards sustainable entrepreneurial behaviour by enhancing entrepreneurs or potential entrepreneurs’ self-efficacy. 

Many kinds of experiences are relevant for entrepreneurs to engage in sustainability and are based in the identification, development and utilization of environmental values [31]. Previous experience allows entrepreneurs to decrease the chances of entrepreneurial failure. Alternatively, this experience would raise entrepreneurs’ confidence and ability to finish specific tasks [10]. Sufficient experience enables greater self-efficacy and supports entrepreneurs in times of crisis, in particular, if entrepreneurs encounter difficulties when implementing strategies on environmental protection or resource recycling. Based on the discussion, the following hypotheses can be derived:

**Hypothesis** **4a.** *The relationship between environmental values and sustainable entrepreneurial intention will be mediated through self-efficacy.*

**Hypothesis** **4b.** *The relationship between environmental values and self-efficacy of entrepreneurs or potential entrepreneurs will be positively moderated by the level of experience.*

## 3. Data and Methods

To test these hypotheses, we employed a meta-analysis method as an effective tool to merge existing empirical studies, to aggregate and to process the independent research results from these studies. This method also allows researchers to evaluate the total correlation between variables. The application of a meta-analysis has some tradition in the entrepreneurship literature [32]. Then, we tested the moderators of attitude, social norms and self-efficacy with respect to the main relationship (environmental values—sustainable entrepreneurial intention). Finally, we utilized results from empirical studies to draw some managerial and theoretical implications. 

The structure of this part is as follows: Firstly, we discuss the retrieval range and the coding standards, and then the sample characteristics are discussed. Secondly, the meta-analytic procedure is described. Thirdly, the meta-analysis is applied to calculate the correlation of the main relationship, with respect to the main effect size and with regard to testing the publication bias. Thirdly, the structural equation model analysis is used to test the mediating effects of the three predictor variables (personal attitude, social norm and self-efficacy) on the key relationship (environmental values to sustainable entrepreneurial intention). Lastly, the meta-analysis was used to test the moderating effects of the control variables with respect to the key relationship. 

### 3.1. Selection of Studies and Code of Variables

**Figure 1:**
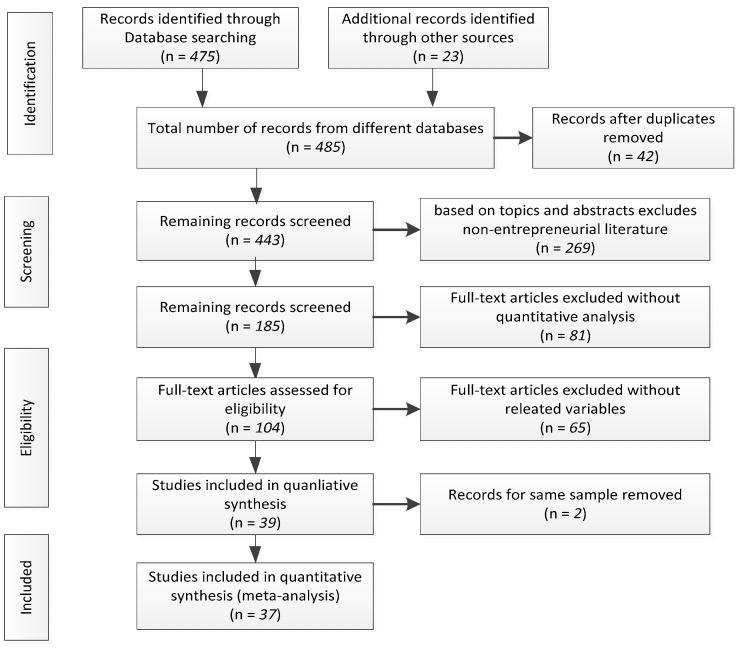
Flow diagram (article selection process).

In order to identify the sample of published and unpublished articles discussing the main relationship between environmental values and sustainable entrepreneurial intention, a three-step procedure was followed: Firstly, we reviewed the literature that had been collected from academic databases (Web of Science, JSTOR, SpringerLink, ScienceDirect, EBSCO, Emerald, Wiley Online Library and ResearchGate). We downloaded all relevant references, using combinations of keywords related to sustainable entrepreneurship (e.g., “sustainable start-ups”, “sustainable venture”, “sustainable entrepreneur”), intention (e.g., “intent”, “intention”, “tendency”, “propensity” and “inclination”). Secondly, we searched in the database of SSRN and ProQuest for relevant academic papers and working papers to reduce publication bias. Thirdly, we examined the references of the studies and searched Google Scholar to expand our search range to acquire additional studies. As a result of this procedure, we got 37 articles (an additional conference paper, one additional academic paper and one additional working paper), which led to 40 independent samples with 192,015 observations (removing 2 articles that had the same samples), allowing for 117 effect sizes. A graphical overview of the search process is shown in Figure 1. The studies are discussed in Appendix A.

To improve the reliability of the coding and measurements, the included studies were coded by two coders (the first author and second author both took part in coding the studies). In case of a disagreement, the two authors resolved it through a discussion to determine the results of the coding.

Coding of sustainable entrepreneurship: Sustainable entrepreneurship is different from regular entrepreneurship, as it covers social entrepreneurship, environmental entrepreneurship and economic entrepreneurship [33]. Sustainable entrepreneurship is interpreted as entrepreneurial activities to realize social, environmental and economic values by discovering, creating, evaluating and exploiting opportunities to offer sustainable products or services [34]. Sustainable entrepreneurship, green entrepreneurship and eco-entrepreneurship are to some extent used synonymously [5]; these terms are always overlapping, which makes it hard to distinguish clearly [35]. Regular entrepreneurship mainly focuses on economic goals, while the main goal of sustainable entrepreneurship is creating sustainable development through starting a new venture by entrepreneurial activities [36], and entrepreneurs should have sustainable intention to achieve sustainable entrepreneurship [37]. Entrepreneurial intention reflects a person’s ambition to take part in entrepreneurial activities. Intention and propensity are interchangeable and synonyms, as well as inclination, intent, tend and tendency. Therefore, we searched the related literature by using keywords of sustainable entrepreneurship, entrepreneurship and sustainability and entrepreneurship with sustainable development; green entrepreneurship eco-entrepreneurship and ecological entrepreneurship; and combining the keywords intention, intent, inclination, propensity, proclivity, tend and tendency.

Coding of environmental values: Environmental values are mainly reflected in environmental protection and application. First, we coded the environmental values by measuring the values attached to issues related to the dimensions of “people–profit–planet” [38,39]. This category stands for values attached to environmental issues. It contains harmless products/service, environmentally responsible materials, adoption of green technologies, environmental care, eco-efficiency, eco-design, development of sustainable technologies or environmental maintenance [38]. Secondly, we coded the category that is related to green values [19,20], environmental values [2] and biospheric values [9], including respecting the earth, unity with nature, preventing pollution and protecting the environment [9]. Thirdly, we coded a category that in a way reflects environmental issues, for instance, environmental protection [40], pro-environmental behaviour [41], shrinking population [27], waste reduction [13] and environmental practices [13]. The values are identified as the stable guiding codes of people’s lives, which can support attitudes and behaviours [10]. Therefore, when scholars measured sustainability orientation, preferring environmental values rather than social responsibility under the green, environmental and ecological entrepreneurship context, we also code it as environmental values with respect to environmental protection, being environmentally oriented, environment performance or environment problems [29]. In case one variable measures environmental and social values together, we selected it as environmental values if this variable has more environmental factors than social factors; thus, the percentage of environmental factors is more than 50%. Fourth, we coded a category with respect to other environmental factors: it contains waste recycling, growing trees and flowers, non-environmental pollution, sewage disposal system, preventing the loss of resources, durable products, use of renewable energy sources and agricultural land and obtaining international organization for standardization certification [42]. 

Coding of attitude: Attitude reflects a person’s favourable evaluation of a particular behaviour [43]. It is a key influential factor of pro-environmental behaviour and sustainable entrepreneurial intention [7]. We used specific keywords to code it, including sustainability attitude [27], attitude towards the behaviour [9,17], attitude to sustainable entrepreneurship [2] and entrepreneurial attitude [7].

Coding of social norms: Social norms refers to the assessment of social pressure to perform or not perform a behaviour of sustainable entrepreneurship [1]. Social norms and subjective norms have been used synonymously in previous studies [7]. Social norms refer to individuals’ perception of social norms, such as personal norms [41]. Social norms are also a predictor for sustainable entrepreneurial intention; we coded it by searching for terms like “social norms”, “subjective norms” or “personal norms”.

Coding of self-efficacy: Self-efficacy is a key predictor for sustainable entrepreneurial intention; it refers to the level of entrepreneurs or potential entrepreneurs’ confidence and ability to undertake a certain activity [10]. To some extent, sustainable entrepreneurship overlaps with the perceived feasibility [43] and the perceived behavioural control [17]. We used certain specific keywords to code it, including “self-efficacy”, “entrepreneurial self-efficacy”, “perceived feasibility” and “perceived behavioural control”. 

Coding of experience: Experience mainly refers to the knowledge from entrepreneurs or potential entrepreneurs when figuring out environmental values and sustainable entrepreneurship. To obtain more data, we employed a subjective coding standard: if more than 50% of the survey responders are entrepreneurs, we defined it as high-level experience (HE = 1); if more than 50% of the survey responders are students without entrepreneurial experience, we defined it as low-level experience (LE = 0). 

### 3.2. Meta-Analytic Procedures

First, we performed meta-analytic procedures by following ideas developed by Stam et al. [32] and Getzner et al. [26]. We took Pearson product–moment correlations as our effect sizes. When the correlation was not reported, we used other statistics (e.g., *t*-test, regression coefficient) instead, and then converted them into correlations using the conversion formula put forward by Rosenthal [44]. When studies reported multiple correlations between given values and sustainable entrepreneurs’ intention, we aggregated them by calculating the mean value. When studies reported the same samples in different articles, we selected the earlier one. For instance, Koe et al. [45,46] and Majid et al. [47] all used the same sample for their study about intention towards sustainable start-ups; we chose the sample of Koe et al. [45], which contained the earlier analysis. 

In order to estimate the overall relationship between the driving values of environment and sustainable entrepreneurial intention, we calculated all the studies’ weighted average effect size. We corrected all variables for measurement unreliability. We adopted the funnel filling method to test for a potential publication bias; the results were relatively symmetric, indicating that there was no serious publication bias; the funnel plots are shown in Appendix B. In addition, we also assessed the potential publication bias by the file-drawer method, which calculates the fail-safe N (5k + 10, k is the number of studies), which is the number of unpublished studies. If the result is calculated as zero, it would render each effect size as statistically insignificant. As shown in Table 1**,** all fail-safe N was more than the values of 5k + 10. For example, the overall relationship between environmental values and sustainable entrepreneurial intention was 1188; it exceeded the threshold value of 75. Our three-step procedure returned 37 articles (two articles were not published); it was unlikely that 1188 unpublished studies remained undetected. Therefore, although publication bias could not be completely ruled out, it was not a serious issue.

Next, we calculated the 95% confidence intervals of the sample-size weighted mean correlation; if the 95% confidence interval of a given effect size did not include zero, the statistics of the considered effect size was significant at α = 0.05 level. We also computed I-square to test for heterogeneity in the relationship in our study. High I-squared values would show that the variability of studies is due to heterogeneity, and this is not by chance. If the I-squared values exceeded 75%, the *p*-value is less than 0.1; this may be a high heterogeneity. If the effect size of the selected studies is significant, the random effect model will be selected. If the effect size is insignificant, a fixed random model was selected. To test our hypotheses, we calculated the moderator’s effect sizes and the I-squared values. As we were undertaking the analysis across different levels of moderators, a random effects model was used to combine the studies within each subgroup and yield the overall effect. We selected a random-effects analysis, comparing the effects at different levels of moderators. If the I-squared values tend to decrease, the moderator may affect the meta-analytical distribution; therefore, the given relationship becomes more affected due to the homogeneity of the effects of the moderators. 

## 4. Results 

This section discusses some of the results by calculating the main effects, mediating effects and moderating effects through a meta-analysis and the structural equation model.

### 4.1. Main Effects of Environmental Values on Sustainable Entrepreneurial Intention

Table 1 reported the meta-analytic results for the main relationship between environmental values and sustainable entrepreneurial intention. The main relationship is positive and significant (r = 0.311, *p* < 0.001). **H1a** is supported. The sample size can be considered as moderately large [32]. The effect size is statistically significant for the reason that the 95% confidence interval did not include zero (it ranges from 0.209 to 0.407). The I-squared of all effect sizes was more than 75%. There was high heterogeneity, therefore a random effect model was chosen. 

**Figure 2:**
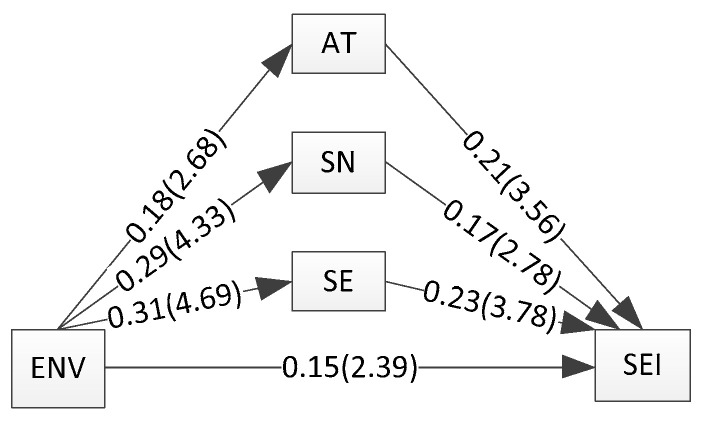
Structural equation model. Notes: ENV = environmental values; SEI = sustainable entrepreneurial intention; AT = attitude; SN = social norms; SE = self-efficacy.

The correlations of three factors influencing the main relationship are also reported in Figure 2. The confidence interval of every effect size did not include zero except the effect size of environmental values to attitude (r = 0.182, *p* > 0.05) which was not statistically significant. Comparing with social norms and self-efficacy, the effect size of environmental values to social norms (r = 0.286, *p* < 0.001) was significantly larger than the effect size of environmental values to self-efficacy (r = 0.308, *p* < 0.001). The values of the correlation coefficients differed very little. The effect size of self-efficacy towards sustainable entrepreneurial intention (r = 0.436, *p* < 0.001) was significantly larger than the other influence factors (attitude, r = 0.392, *p* < 0.001; social norms, r = 0.368, *p* < 0.001).

### 4.2. Mediating Effects of Environmental Values on Sustainable Entrepreneurial Intention

To test whether the relationship between environmental values and sustainable entrepreneurial intention would be mediated by attitude, social norms and self-efficacy, we introduced a two-step procedure to deal with this issue. In the first step, we calculated all correlations among the environmental values, attitude, social norms, self-efficacy and sustainable entrepreneurial intention by supplementing the remaining three correlations of each relationship in attitude, social norms and self-efficacy from our selected samples. Furthermore, we used the harmonic mean to calculate the total sample size. In a second step, we used the effect sizes from the meta-analysis in the mediating test in structural equation model analysis software, and then constructed a structural equation model (see Figure 2). Table 2 and Table 3 report the mediating results for the relationship between the environmental values and sustainable entrepreneurial intention. Before discussing the mediators, the direct result of the main relationship is significant and positive. Afterwards, we added the mediators (attitude, social norms and self-efficacy) into the relationship. All factors (attitude, social norms and self-efficacy) have mediating effects on the main relationship. Compared to the difference of the mediating effects from previous versus later data, the effects of the later data are significantly smaller than from previous data (main effects reduce from 0.310 to 0.150). This shows that the mediators have a significant effect. Thus, **H2a, H3a** and **H4a** are supported.

### 4.3. Moderating Effects of Environmental Values on Sustainable Entrepreneurial Intention

To test whether (or not) the moderating effects are present, we compared the effects of high-level experience and low-level experience among the relationship between environmental values and sustainable entrepreneurial intention. The results are shown in Table 4. Experience promotes the influence of environmental values on sustainable entrepreneurial intention. Furthermore, environmental values will be more positively related to sustainable entrepreneurial intention for entrepreneurs or potential entrepreneurs with low-level experience (r = 0.321, *p* < 0.001) compared to those entrepreneurs with high-level experience (r = 0.288, *p* < 0.01). **H1b** is therefore supported. High-level experience will not moderate the relationship between environmental values and attitude (r = 0.228, *p* > 0.05), nor low-level experience (r = 0.135, *p* > 0.05). **H2b** is not supported. The high-level experience will not influence the link between ENV and social norms (r = 0.195, *p* > 0.05); on the opposite, low-level experience will influence the relationship between them (r = 0.291, *p* < 0.01). **H3b** is not supported. The main relationship will be moderated by experience, and environmental values will be more positively related to sustainable entrepreneurial intention under high-level experience (r = 0.385, *p* < 0.001) than low-level experience (r = 0.212, *p* < 0.001). **H4b** is supported. 

## 5. Discussion and Implications

This section summarises the key results relevant to environmental values and sustainable entrepreneurial intention, discussing the reason why these relationships existed, and suggests some conclusions for the formation of sustainable entrepreneurial intention by entrepreneurs or potential entrepreneurs. 

### 5.1. Discussion

After a series of analyses, we could generate some results as shown in Table 5. Despite the increase in studies examining the role of environmental values implementing sustainable entrepreneurship, it is still necessary to further study the factors influencing the intentions for sustainable entrepreneurship, and how these intentions can be promoted by environmental values influencing sustainable entrepreneurs’ intention. This line of research can support entrepreneurial behaviour to become more sustainable. Most literature has been published rather recently (within the past three years); however, sustainable entrepreneurship seems to attract the attention of a wider audience of scholars. 

Our analysis of independent samples indicates that there is a positive relationship between environmental values and sustainable entrepreneurial intention (r = 0.311). This finding is vital for entrepreneurs or potential entrepreneurs. It is linked to previous studies, such as by Nuringsih et al. [19], proposing that green value encourage sustainable entrepreneurship, while the results differ from the study by St-Jean et al. [10]. Green marketing and green producing among other things based on environmental values, could inspire entrepreneurs to create and produce environmental products and services [19]. Green marketing allows people to capture green opportunities by enhancing the personal intention to start a sustainable enterprise. Sometimes, industry standards link environmental problems to social responsibility [10], but at the social level makes it becomes difficult for entrepreneurs to set up a new sustainable enterprise. As a result, the intention for sustainable entrepreneurship will decrease. Although economic value has attracted the attention of entrepreneurs, an increasing number of sustainable entrepreneurs focused on greening the bottom line of their company to deal with environmental issues [33]. Our findings will encourage entrepreneurs or potential entrepreneurs to form behavioural intention based on environmental values that could generate entrepreneurial opportunities to create a sustainable enterprise. These enterprises could rely on environmental sustainability to develop innovative behaviour; it could bring new opportunities to the market, and strengthen environmental values [12]. Relatively speaking, the realization of social values and economic values tends to be a firm-level issue, while environmental values are more like macro-level issues related to global problems. However, it increasingly becomes more important to attach greater weight to the effects of environmental values. A new understanding of environmental values will have a positive impact on the environment and the natural resource currently wasted, which could promote cleaner production. This finding helps to enhance entrepreneurs’ intentions to become sustainable entrepreneurs, as predicted by their behaviour towards sustainable entrepreneurship [2]. 

The relationship between environmental values and sustainable entrepreneurial intention is moderated by the level of experience (HE: r = 0.321, *p* < 0.001; LE: r = 0.288, *p* < 0.01). This surely is an interesting finding on its own. Both high-level and low-level experiences are positively correlated with the relationship between environmental values and sustainable entrepreneurial intention. The variable for environmental values is positively related to sustainable entrepreneurial intention of entrepreneurs or potential entrepreneurs with high-level experience compared to those entrepreneurs with low-level experience. This is consistent with Sardianou et al. [48], where many entrepreneurs with experience were willing to take eco-friendly actions and environmental awareness was positively correlated to entrepreneurs or potential entrepreneurs’ intention to adopt sustainable behaviour or practices [48]. Environmental improvements should rely on policies related to learning from experience, as high-level experience promoted entrepreneurs into the beliefs that implementing sustainable entrepreneurs would be better, which could enhance entrepreneurs’ intention to set up a sustainable enterprise. It also is the similar to the idea developed by Kuckertz et al. [29], in that low-level experience on environmental issues is more likely to be translated into entrepreneurial intention compared to potential entrepreneurial behaviour; it also might be concerned with the individual itself [29], for example, the influence of extrinsic rewards and intrinsic rewards [2]. Entrepreneurs with high-level experience could be exposed to ethical situations in which they act irresponsible, and then they are less likely to form an intention to adopt sustainable entrepreneurial behaviour [40]. This is also consistent with the view of Koe et al. [45]: the intention towards sustainable entrepreneurship mainly focuses on the intention rather than actual action. Many potential entrepreneurs are college students who have not graduated or are fresh graduates. They usually have no or hardly any low-level entrepreneurial experience, only receiving courses related to entrepreneurship. In our study, entrepreneurs with low-level experience were more likely to increase their intention to become sustainable entrepreneurs than those with high-level experience. A close reading of the literature reveals that there are two possible approaches. For entrepreneurs with high-level experience, their core goals of enhancing intention towards sustainable entrepreneurship is to build a new venture successfully and to take a series of sustainable practices [12]. While, for those with low-level experience, their main goals have been to start the behaviour of sustainable entrepreneurship. High-level experience, which mainly came from start-ups, did contribute to entrepreneurs to achieve a transformation of their values into specifying intentions, while low-level experience, which mainly came from college courses, might provide help to potential entrepreneurs to gain some knowledge about environmental values, and then generate incentives for sustainable entrepreneurship. Without enough experience, potential entrepreneurs might have sufficient emotions about environmental values that will drive them towards entrepreneurial behaviour related to sustainability. So, entrepreneurs with high-level experience give more attention to feasibility than those with low experience. Considering the feasibility of sustainability, these entrepreneurs or would-be entrepreneurs are more likely to engage in low-tech entrepreneurial activities of small and medium enterprises [43,45]; for instance, in manufacturing, construction and agriculture, in which it is more expensive to implement sustainable practices than in high-tech industries. From these findings, we derived the conclusion that the overall benefit of experience is that entrepreneurs with low-level experience will be more positively related to the relationship between environmental values and sustainable entrepreneurial intention than those with high-level experience.

The relationship between environment value and sustainable entrepreneurial intention is mediated through attitude towards sustainable entrepreneurship (mediating effect = 0.031, *p* < 0.001). However, this experience is not acting as a moderator in the relationship between environmental values and sustainable entrepreneurial intention. Environmental values has a positive impact on attitude towards sustainable entrepreneurship [9]. Entrepreneurs who possess a positive attitude towards sustainable entrepreneurship have much more of an intention to start up a sustainable venture [43]. A positive attitude helps entrepreneurs to enhance the balance of values related to people–profit–planet, which leads to achieving sustainable entrepreneurship [38,43]. Entrepreneurs or potential entrepreneurs worry about spending time and resource on environmental values, which would increase the risks of starting a business without generating any revenue. While a positive attitude makes them believe that the implementation of environmental values would promote sustainable entrepreneurship, especially successful sustainable entrepreneurship, it might also wipe out concerns about business failure [2]. No matter which level experience of entrepreneurs have, with a positive attitude, the level of tolerance of entrepreneurial failure raises. A positive attitude enables entrepreneurs to face various possible difficulties in a way that allows a new venture to start that adds entrepreneurs’ intention to sustainable entrepreneurship. However, a negative attitude may fade away the intention for the fear of business failure. Now, the attitude has a positive mediating effect on the relationship between environmental values and sustainable entrepreneurial intention. The more importance on the environmental values entrepreneurs give, the more of an active attitude of entrepreneurs will have on entrepreneurship, in general. Experience would not moderate the relationship between environmental values and attitude towards sustainability positively. Although the hypothesis was not supported, the results are meaningful. In the context of developing environmental value, experience would not promote entrepreneurs’ attitude to be positive; maybe it exist among other variables moderating this relationship: on the one hand, the entrepreneurs’ attitude was influenced by perceived support and perceived barriers [49], on the other, the detrimental effect of the entrepreneurs’ experience come from their internal and external influences [29], for instance, whether subjects had been abroad, where different countries with different policies affected entrepreneurs in their decisions. 

The relationship between environment value and sustainable entrepreneurial intention is mediated through social norms (mediating effect = 0.046, *p* < 0.001). Environmental value is more positively related to the social norms of entrepreneurs or potential entrepreneurs with low-level experience (r = 0.291, *p* < 0.01) than to those with high-level experience (r = 0.261, *p* > 0.05). So, the relationship between values of environment and social norms will be negatively moderated by the studies that have confirmed that social norms are positively related to sustainable entrepreneurial intentions [12,17]. Previous studies have already found that social norms influence sustainable entrepreneurial behaviour [1]. As society pays more attention to environmental protection, the pressure of social norms will drive entrepreneurs or potential entrepreneurs to pursue sustainable environmental development. This is in line with Rezai et al. [13]. Previous studies about enterprises accepting environmental norms have shown that enterprises could generate economic value [13]. Social norms promote the gradual transformation of an enterprise’s production process into a process for realizing value based environment protection, and social norms can be regarded as a predictor of sustainable entrepreneurial propensity [43]. Such situations drive entrepreneurs to start an environmental value-oriented venture and contribute to the formation of an intention for sustainable entrepreneurial behaviour. The value attached to the natural environment increases with the development of society when developing a start-up company. Based on the influence of the institutional environment, an increase in the value attached to the environment will involve an increase of social norms [13]. The changes of social norms promote the relationship between environmental values and sustainable entrepreneurship indirectly. Surprisingly, high-level experience does not affect the relationship of environmental values to social norms; however, low-level experience actually does generate such an effect. Under the pressure of conformity, entrepreneurs or potential entrepreneurs with low-level experience, especially young adults, tend to imitate other entrepreneurs. To some extent, social norms help environmental values to be determined unconsciously, and environmental values constitute a code of conduct, which positively influences its initiatives towards sustainable entrepreneurship [23]. 

The relationship between environment values and sustainable entrepreneurial intention is mediated through self-efficacy (mediating effect = 0.060, *p* < 0.001). Environmental values will be more positively related to the self-efficacy of entrepreneurs or potential entrepreneurs with high-level experience (r = 0.385, *p* < 0.001) than those with low-level experience (r = 0.212, *p* < 0.001). The result of the mediator is similar to the view of Ahmed et al. [50]. From the results, we find that all effect sizes were greater than zero. Entrepreneurs or potential entrepreneurs with a high self-efficacy have more confidence to pursue values set in the environment without self-doubt; they believe in themselves and seek opportunities in a situation of competitive pressure, which allow them to become sustainable entrepreneurs [50]. Entrepreneurs’ beliefs in their abilities to succeed in start-ups can promote the enhancement of their intention of taking advantage of environmental protection and value creation [2]. Much confidence come with self-efficacy that could make entrepreneurs believe that paying more attention to the values of the environment at the beginning could enhance sustainable entrepreneurial opportunities, as well as environmental performance [50]. In addition, high-level experience helps to raise entrepreneurs’ comprehensive abilities, and increased self-efficacy helps entrepreneurs to identify opportunities for developing environmental sustainability. Experience usually provides some attraction towards more general entrepreneurship and the generation of economic value compared to improvements in the self-efficacy of entrepreneurs [18]. Sufficient experience enables entrepreneurs to recognize the importance of environmental value. Green practices make it easier for entrepreneurs to develop sustainability with high-level self-efficacy [13]. Therefore, entrepreneurs with high self-efficacy are confident that they could figure out a way on how to implement environmental values; it makes entrepreneurs’ intention towards sustainable entrepreneurial behaviour more practical. 

### 5.2. Implications

These findings are of great importance as we have shown that environmental values have a positive impact on sustainable entrepreneurial intention. These values stimulate entrepreneurs or potential entrepreneurs in a way that enhances the intention for sustainable entrepreneurship. Attitude, social norms and self-efficacy are variables that serve as links between the relationship of environmental values to sustainable entrepreneurial intention. Internal and external drives are adopted to improve entrepreneurs or potential entrepreneurs’ recognition of environmental value creation, and the mediating effects of experience on attitude, social norms and self-efficacy directly promote the enhancement of intention to sustainable entrepreneurship, so as to predict sustainable entrepreneurial behaviour. In the following, we provide some suggestions for entrepreneurs or potential entrepreneurs who intend to become sustainable entrepreneurs. 

Firstly, the importance of environmental values does increase the willingness of entrepreneurs to become sustainable entrepreneurs. The government, social organization and other institutions can publicize the advantages of implementing environmental protection, green technology and cleaner production, and use the typically successful entrepreneurial enterprises to guide entrepreneurs or potential entrepreneurs to enhance environmental values among entrepreneurs. In addition, we encourage the government to formulate preferential policies for entrepreneurs to implement values regarding the environment, and encourage enterprises to innovate green technology and cleaner production. For entrepreneurs with low-level experience, especially college students, relevant education of environmental protection, resource conservation and energy efficiency in entrepreneurship courses should be used to guide their positive views on the values of the environment, the people–profit–planet’s sustainability and the importance of cleaner production. While, for entrepreneurs with high-level experience, preferential entrepreneurial policies should be used to attract entrepreneurs to learn the ways and methods of implementing values created from environment efficiently. 

Secondly, attitude is influenced by attaching importance to environmental values: the more efficiently environmental values are playing into practices, the more likely it is to stimulate attitudes to become more positive towards sustainable entrepreneurship. A too pessimistic attitude is not conducive to the formation of sustainable entrepreneurial intention; entrepreneurs are afraid of gains and losses, and conservatives are more inclined to rapidly gain profits in entrepreneurship. Entrepreneurs with a positive attitude tend to be risk-oriented and rely on environmental sustainability to achieve sustainable economic, social and environmental development. The publicity of environmental practices should be vigorously promoted to guide entrepreneurs to have a positive attitude, correctly recognize the role of green implementation and stimulate entrepreneurs to pursue sustainable entrepreneurship. The channels of environmental protection, green technology, low consumption and high yield results are what entrepreneurs need to pursue. Less positive entrepreneurs may have intent to choose environmental practices with less risk and mature technology, and imitating innovation may be green implementing’s best strategy. Risk-orientated entrepreneurs with a positive attitude are suggested to choose ecological innovation strategies to make development decisions for value creation of green efficiently. 

Thirdly, wide distribution of propaganda and practice methods by the government, universities and society about environmental values, to some extent, can stimulate entrepreneurs to perceive larger social norms. Entrepreneurs with high-level experience could certainly influence entrepreneurs with low-level experience by the sustainability practices of environmental values, especially for potential entrepreneurs without entrepreneurial experience as to the formation of social norms. We encourage the government to set different implementation of environmental values according to different industries, for the formation of high-level actual social norms. For entrepreneurs or potential entrepreneurs with low-level experience, they can enhance their intention to implement sustainable entrepreneurship by taking part in entrepreneurial projects competitions and adopting imitation of successful enterprises. That is to say, the society should take some measures to make both perceived social norms and actual social norms together to be high level, which helps to form a standard code of conduct for entrepreneurship. For example, a business incubator should carry out relevant training regularly. The government should jointly issue relevant policies, form basic codes of conduct and implement the reward and punishment system. Universities should also open the relevant courses to form a better sustainable entrepreneurship ideology.

Fourthly, high-level experience is more likely to promote the improvement of entrepreneurs’ self-efficacy, and it is believed that they can fully exploit environmental values, so as to achieve comprehensive and sustainable entrepreneurial behaviour. In this initiative to improve the function of the business incubator, encourage the government to introduce more preferential policies for entrepreneurship, such as the mechanism of trial and errors. With the help of large to small enterprises, mature enterprises to new organizations, entrepreneurs’ personal entrepreneurial ability will be improved, and more places are provided for entrepreneurs to continuously try to efficiently obtain environmental values. Considering the cost of sustainable entrepreneurship implementing, we recommend that the business incubator can give more time and resource for sustainable entrepreneurs than that for general entrepreneurs, and encourage them to have the sustainable business intent.

## 6. Conclusions, Limitations and Outlook

This study has sought to respond to the relation of environmental values and sustainable entrepreneurial intention, to carry out studies in greater depth for exploring the link of some variables (attitude, social norms and self-efficacy) to the main relationship. The results of these research studies were applied in a meta-analysis method and structural equation model analysis method to achieve our research goals. In the end, six hypotheses are supported, and two hypotheses are not supported. Values of the environment are positively and significantly correlated to sustainable entrepreneurs’ intention; the variables attitude, social norms and self-efficacy serve as mediators, positively and significantly influencing the main relationship; values of the environment will be more positive to sustainable entrepreneurs’ intention for entrepreneurs with high-level experience than those with low-level experience. However, the relationship between values of the environment and an entrepreneur’s attitude to sustainable entrepreneurship would not be moderated by experience; experience was negatively related to environmental values and social norms. Experience could promote the relationship between environmental values and self-efficacy positively, while values of environment would be more positively related to the self-efficacy of entrepreneurs or potential entrepreneurs with high-level experience than those with low-level experience. All these findings help entrepreneurs or potential entrepreneurs to enhance their intention to sustainable entrepreneurship, and some advice is put forward.

However, this study also has three limitations, which are as follows. First, subjective variables (social norms, environmental values) initiate the chain of effects that affect the action variables (self-efficacy, sustainable entrepreneurial intention) [23], and attitude influences social norms [1]. How these variables influence each other, it still deserves to further study. Secondly, this study mainly uses a meta-analysis to test the influences of the three factors on the relationship between environmental values and sustainable entrepreneurial intention. How these variables and findings influence values of the environment, intention to sustainable entrepreneurship and sustainable entrepreneurial behaviour, also deserves to be studied in depth. Thirdly, sustainable entrepreneurship focuses on social values, economic values and environmental values; whether the three factors mentioned above show the same influences, and which value is more likely to drive the formation of sustainable entrepreneurs’ intention, would be meaningful for us to solve.

Based on these three limitations, we will do a further study about the different influences of social values, economic values and environmental values to sustainable entrepreneurial intention and sustainable entrepreneurial behaviour. Direct and indirect influences of different predictors towards sustainable entrepreneurial intention and behaviour also need to be researched in depth. The development of sustainable entrepreneurship, including “people–profit–planet”, helps enterprises to make a profit, helps to solve social problems, protect the natural environment and ensures resource recycling and regeneration. Compared with other types of entrepreneurship, sustainable entrepreneurship is more conducive to the sustainable development of an enterprise, a country or even the globe. Currently, the implementation of sustainable entrepreneurial behaviour comes from the increase of sustainable entrepreneurial intention. Studies on sustainable entrepreneurship are increasing, but there still exist research gaps. We expect that there will be more studies related to sustainable entrepreneurship in the future.

## Figures and Tables

**Table 1:** Results of the meta-analysis on the main effects.

Variable	k	N	r	95% CI	*p*	I	Fail-Safe N
ENV-SEI	13	3823	0.311	(0.209, 0.407)	0.000	91.227	1188
ENV-AT	8	7660	0.182	(−0.016, 0.367)	0.071	97.966	299
ENV-SN	5	1441	0.286	(0.135, 0.424)	0.000	89.077	156
ENV-SE	5	1911	0.308	(0.159, 0.443)	0.000	91.411	264
AT-SEI	19	181,861	0.392	(0.276, 0.497)	0.000	98.294	5134
SN-SEI	14	179,721	0.368	(0.208, 0.510)	0.000	98.636	3032
SE-SEI	20	181,203	0.436	(0.306, 0.551)	0.000	98.584	8991

Notes: ENV = environmental values; SEI = sustainable entrepreneurial intention; AT = attitude; SN = social norms; SE = self-efficacy; k = number of samples; N = number of observations; r = reliability corrected and sample size weighted effect size; %variance = sampling error variance (unit = %); 95%CI = 95% confidence intervals; *p* = statistic based on the test for a significant degree of difference in effect sizes, from a two-tailed test; I = the I-squared of heterogeneity; Fail-safe N = the number of missing studies that would bring the *p*-value to >alpha.

**Table 2:** Results of the direct effect among the constructs and proposed hypotheses.

Constructs	Direct Effect	Result
ENV→SEI	0.310 ***	Supported

Notes: *** *p* < 0.001.

**Table 3:** Results of the mediating analysis among the constructs and proposed hypotheses.

Constructs	Mediated Effect/Indirect Effect	Result
ENV→SEI	0.150 ***	Supported
ENV→AT→SEI	0.038 *	Supported
ENV→SN→SEI	0.049 *	Supported
ENV→SE→SEI	0.071 **	Supported

Notes: *** *p* < 0.001; ** *p* < 0.01; * *p* < 0.05.

**Table 4:** Results of the moderator effects.

Relationship		r	95% CI	*p*
ENV-SEI	HE	0.288	(0.083, 0.470)	0.007
	LE	0.321	(0.195, 0.437)	0.000
ENV-AT	HE	0.228	(−0.106, 0.516)	0.180
	LE	0.135	(−0.187, 0.430)	0.413
ENV-SN	HE	0.261	(−0.137, 0.586)	0.196
	LE	0.291	(0.098, 0.464)	0.004
ENV-SE	HE	0.385	(0.197, 0.546)	0.000
	LE	0.212	(0.099, 0.319)	0.000
AT-SEI	HE	0.315	(0.137, 0.473)	0.001
	LE	0.448	(0.294, 0.579)	0.000
SN-SEI	HE	0.386	(0.112, 0.606)	0.007
	LE	0.343	(0.243, 0.437)	0.000
SE-SEI	HE	0.517	(0.257, 0.707)	0.000
	LE	0.381	(0.259, 0.491)	0.000

**Table 5:** The overall results of environment value and sustainable entrepreneurial intention.

Number	Hypothesis	Results
**H1a**	Environmental value will be positively related to sustainable entrepreneurial intention.	Supported
**H1b**	The relationship between environment value and sustainable entrepreneurial intention will be positively moderated by the level of experience.	Supported
**H2a**	The relationship between environment value and sustainable entrepreneurial intention will be mediated through attitude to sustainable entrepreneurship.	Supported
**H2b**	The relationship between environment value and the attitude of entrepreneurs or potential entrepreneurs will be positively moderated by the level of experience.	Not supported
**H3a**	The relationship between environment value and sustainable entrepreneurial intention will be mediated through social norms.	Supported
**H3b**	The relationship between environment value and the social norms of entrepreneurs or potential entrepreneurs will be positively moderated by the level of experience.	Not supported
**H4a**	The relationship between environment value and sustainable entrepreneurial intention will be mediated through self-efficacy.	Supported
**H4b**	The relationship between environment value and the self-efficacy of entrepreneurs or potential entrepreneurs will be positively moderated by the level of experience.	Supported

**Figure A1:**
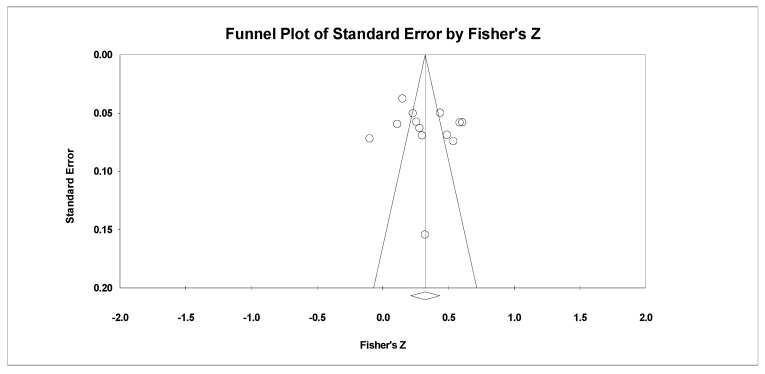
Funnel plot of ENV-SEI.

**Figure A2:**
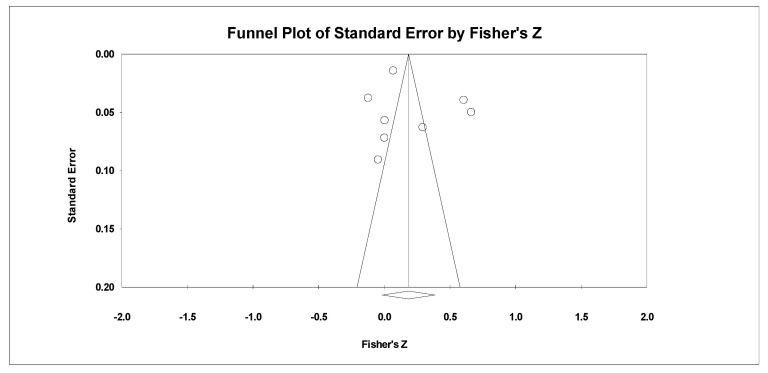
Funnel plot of ENV-AT.

**Figure A3:**
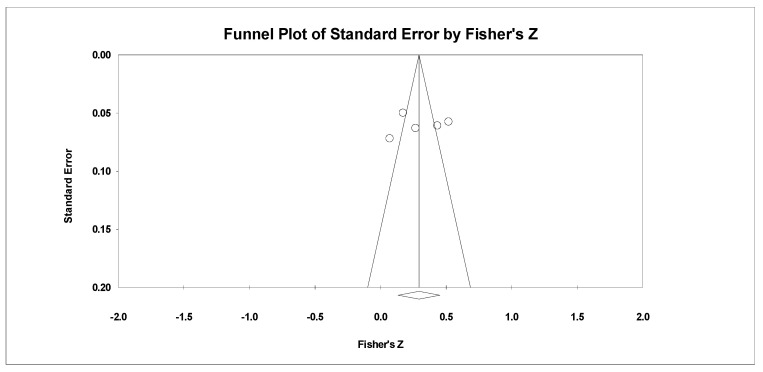
Funnel plot of ENV-SN.

**Figure A4:**
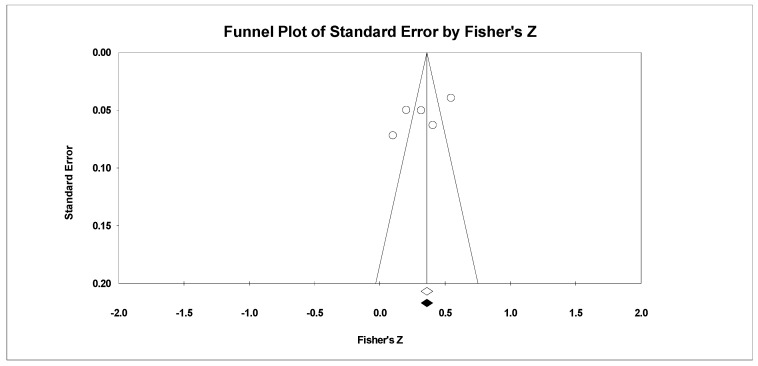
Funnel plot of ENV-SE.

**Figure A5:**
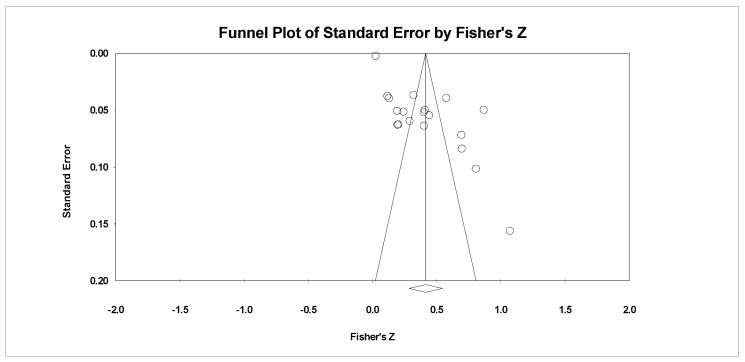
Funnel plot of AT-SEI.

**Figure A6:**
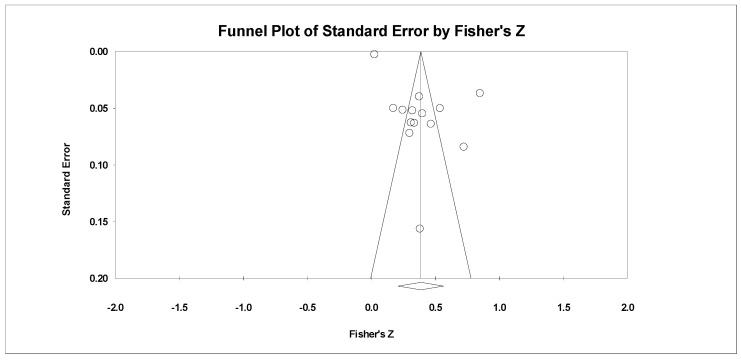
Funnel plot of SN-SEI.

**Figure A7:**
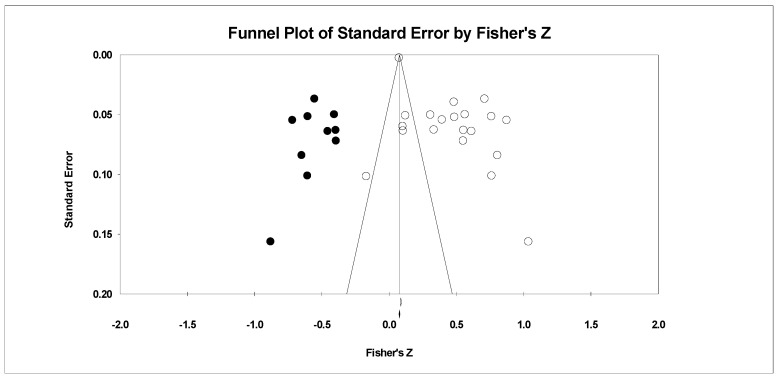
Funnel plot of SE-SEI.

## Data Availability

Data of the retrieved studies are shown in Appendix A.

## Appendix A

**Table A1:** Overview of studies included in the meta-analysis.

Author (Year)	N	Pub	Ex	Country	ENV-SEI	ENV-AT	ENV-SN	ENV-SE	AT-SEI	SN-SEI	SE-SEI
Abina (2015) [14]	344	yes	0	Nigeria							0.373
Ahmad (2015) [30]	100	yes	0	Malaysia							−0.169
Ahmed (2020) [50]	401	yes	0	Malaysia				0.307			
Alamineh (2019) [7]	145	yes	0	Ethiopia					0.602	0.618	0.667
Cavazos-Arroyo (2016) [18]	745	yes	1	Mexico					0.310	0.690	0.610
Fatoki (2019) [51]	301	yes	1	South Africa	0.541						
Ghazali (2019) [41]	307	yes	0	Malaysia			0.477				
	274		0	China			0.409				
Koe (2014a) [43]	249	yes	1	Malaysia					0.381	0.434	0.545
Koe (2014b) [45]	404	yes	1	Malaysia					0.387	0.490	
Koe (2019) [52]	185	yes	0	Malaysia	0.491						
Kuckertz (2010) [29]	712	yes	0	Germany	0.149	−0.123			0.117		
Kuckertz (2019) [21]	212	yes	0	America	0.290						
Kushwaha (2017) [5]	306	yes	0	India	0.249						
Martínez-González (2019) [23]	339	yes	1	Spin					0.416	0.378	0.703
	382		1	Poland					0.238	0.240	0.641
Mbebeb (2012) [1]	44	yes	0	Cameroon					0.790	0.362	0.776
Middermann (2020) [17]	175,280	yes	1	57 countries					0.026	0.025	0.073
Muñoz (2015) [53]	45	yes	1	5 countries	0.310						
Nuringsih (2017) [19]	400	No	0	Indonesia	0.225						
Nuringsih (2019) [20]	300	yes	0	Indonesia	0.526					0.300	0.320
Ploum (2017) [54]	402	yes	0	Netherlands							0.297
Polas (2020) [24]	285	yes	0	Bangladesh					0.282		
Rashid (2018) [55]	101	yes	0	Malaysia							0.642
Rezai (2015) [13]	256	yes	1	Malaysia	0.272	0.284	0.261	0.385	0.193	0.323	0.502
Robina-Ramírez (2019) [56]	5000	yes	1	Spain		0.067					
Saleem (2018) [15]	259	yes	0	Pakistan					0.200		
Sardianou (2016) [48]	287	yes	1	Greece	0.110						
Sargani (2020) [27]	640	yes	0	China					0.129	0.357	
Sher (2020) [2]	650	yes	1	Pakistan	0.580	0.540		0.497	0.520		0.447
Soomro (2019) [22]	284	yes	0	Pakistan							0.100
St-Jean (2018) [10]	197	yes	0	Canada	−0.100	0.000	0.070	0.100	0.600	0.290	0.500
Sudyasjayanti (2017) [6]	100	yes	0	Indonesia					0.667		
Sung (2018) [40]	215	yes	1	Kore	0.453						
Tan (2013) [57]	252	no	0	China, India, Malaysia							0.102
Thelken (2020) [9]	407	yes	0	Netherlands, Germany	0.410	0.580	0.170	0.200	0.700	0.170	0.510
Tiwari (2017) [58]	374	yes	0	India					0.380	0.310	0.450
Vuorio (2018) [31]	393	yes	0	Liechtenstein, Austria, Finland					0.190		0.120
Wagner (2015) [49]	313	yes	0	German		0.002					
	125		1	German		−0.048					

Note: Pub stands for the type of studies publication; yes = published; no = unpublished; ex = experience; high-level experience = 1; low-level experience = 0; a,b and so forth are used to indicate several studies published or unpublished by the same first author in the same year.
